# Clinical Examination of Urine

**Published:** 1871-12

**Authors:** 


*Clinical Examination of Urine.* By Reuben A. Vance, M. D.

In this pamphlet are given some simple practical plans to determine the constituents of urine. In connection with this a description of a convenient apparatus for its speedy analysis.

